# Phytochemicals: Targeting Mitophagy to Treat Metabolic Disorders

**DOI:** 10.3389/fcell.2021.686820

**Published:** 2021-08-03

**Authors:** Zuqing Su, Yanru Guo, Xiufang Huang, Bing Feng, Lipeng Tang, Guangjuan Zheng, Ying Zhu

**Affiliations:** ^1^Guangdong Provincial Hospital of Chinese Medicine, The Second Clinical College of Guangzhou University of Chinese Medicine, Guangzhou, China; ^2^Guizhou University of Traditional Chinese Medicine, Guiyang, China; ^3^The First Affiliated Hospital of Guangzhou University of Chinese Medicine, Guangzhou, China

**Keywords:** phytochemicals, metabolic disorders, mitophagy, mitochondrial dysfunction, oxidative stress, inflammatory response

## Abstract

Metabolic disorders include metabolic syndrome, obesity, type 2 diabetes mellitus, non-alcoholic fatty liver disease and cardiovascular diseases. Due to unhealthy lifestyles such as high-calorie diet, sedentary and physical inactivity, the prevalence of metabolic disorders poses a huge challenge to global human health, which is the leading cause of global human death. Mitochondrion is the major site of adenosine triphosphate synthesis, fatty acid β−oxidation and ROS production. Accumulating evidence suggests that mitochondrial dysfunction-related oxidative stress and inflammation is involved in the development of metabolic disorders. Mitophagy, a catabolic process, selectively degrades damaged or superfluous mitochondria to reverse mitochondrial dysfunction and preserve mitochondrial function. It is considered to be one of the major mechanisms responsible for mitochondrial quality control. Growing evidence shows that mitophagy can prevent and treat metabolic disorders through suppressing mitochondrial dysfunction-induced oxidative stress and inflammation. In the past decade, in order to expand the range of pharmaceutical options, more and more phytochemicals have been proven to have therapeutic effects on metabolic disorders. Many of these phytochemicals have been proved to activate mitophagy to ameliorate metabolic disorders. Given the ongoing epidemic of metabolic disorders, it is of great significance to explore the contribution and underlying mechanisms of mitophagy in metabolic disorders, and to understand the effects and molecular mechanisms of phytochemicals on the treatment of metabolic disorders. Here, we investigate the mechanism of mitochondrial dysfunction in metabolic disorders and discuss the potential of targeting mitophagy with phytochemicals for the treatment of metabolic disorders, with a view to providing a direction for finding phytochemicals that target mitophagy to prevent or treat metabolic disorders.

## Introduction

Autophagy, an evolutionarily conserved catabolic process, degrades intracellular constituents including lipid, glycogen and protein to maintain cellular energy homeostasis in the absence of nutrients (Vargas et al., 2017). In this process, double-membrane vesicles (autophagosomes) will enfold intracellular constituents and then transfer to lysosomes to form autolysosomes where the lysosomal enzyme can degrade the enveloped cargoes. According to the different methods of delivering cytoplasmic components to lysosomes, autophagy can be divided into three different types: macroautophagy (hereafter referred to as autophagy), microautophagy and chaperone-mediated autophagy. Moreover, according to the specificity of the degradation substrate, autophagy can be classified into mitophagy, pexophagy, reticulophagy, ribophagy and xenophagy.

About 50 years ago, autophagy was first described to be triggered to maintain cellular energy balance and cell survival under nutrition-deprived conditions. In the past 10 years, due to the in-depth understanding of the role of autophagy, accumulating study has indicated that autophagy plays a vital role in the physiology and pathology of many diseases, such as metabolic disorders, cancer, Alzheimer’s disease and Parkinson’s disease. Nowadays, it is commonly accepted that autophagy, especially mitophagy, plays a crucial role in the pathology of metabolic disorders such as non-alcoholic fatty liver, type 2 diabetes and metabolic syndrome (Vásquez-Trincado et al., 2016; Sarparanta et al., 2017; Dombi et al., 2018).

Phytochemicals extracted from natural plants have been widely used to treat metabolic diseases including metabolic syndrome (Cicero and Colletti, 2016), type 2 diabetes (Moreno-Valdespino et al., 2020), obesity (Li et al., 2019), insulin resistance (Mahdavi et al., 2021) and cardiovascular diseases (Pop et al., 2018) due to relative safety and multiple beneficial effects. According to an estimation issued by the World Health Organization in 2008, about 80% of diabetic patients rely on herbal medicine (Bacanli et al., 2019).

In view of the fact that more and more phytochemicals are applied to the treatment of metabolic diseases, it is necessary to have a more comprehensive understanding of the effects and potential mechanisms of phytochemicals on metabolic diseases. Therefore, this review will focus on the regulatory mechanisms of mitophagy in metabolic disorders and further explore the potential of targeting mitophagy with phytochemicals for the prevention or treatment of metabolic disorders.

## Mitochondrial Dysfunction and Oxidative Stress

Mitochondrion regulates many physiological functions such as adenosine triphosphate (ATP) synthesis, free radicals generation, fatty acid β−oxidation, calcium homeostasis, and cell survival and death (Liu L. et al., 2020; Kirtonia et al., 2021). Mitochondria are dynamic organelles that can fleetly adapt to changes in cellular energy metabolism by regulating mitochondrial biogenesis, mitochondrial fission, mitochondrial fusion and removal of damaged mitochondria (Vásquez-Trincado et al., 2016; Mills et al., 2017; Keenan et al., 2020). Of note, the uppermost physiological function of mitochondria is to produce ATP via oxidative phosphorylation (OXPHOS) (Bhatti et al., 2017). During OXPHOS, mitochondria will inevitably produce by-product superoxide anions, which can be further converted into reactive oxygen species (ROS) or reactive nitrogen species (RNS) (Kalyanaraman et al., 2018). Under physiological conditions, ROS and RNS act as the regulatory mechanism for cellular redox homeostasis (Liu and Butow, 2006). Mitochondrial ROS, one of the major sources of cellular ROS, consists of hydrogen peroxide (H_2_O_2_), superoxide (O^–^_2_), and hydroxyl (OH), which can impair lipids, proteins and DNA, resulting in mitochondrial dysfunction and cell apoptosis (Kirtonia et al., 2020; Tan et al., 2021). There are various defense systems to counter ROS-induced oxidative stress, such as catalase (CAT), superoxide dismutase (SOD) and glutathione peroxidase (GSH-PX). However, high levels of ROS will unduly oxidize lipids, DNA and proteins, thereby destroying cell membranes and other cellular structures. In general, mitochondrial ROS can damage mitochondrial DNA to disturb the physiological functions of mitochondria. And the accumulation of damaged mitochondria and the overexpression of mitochondrial ROS will reinforce each other, forming a vicious cycle, and eventually lead to mitochondrial dysfunction. Accumulating evidence has shown that oxidative stress is involved in various pathological conditions including insulin resistance, type 2 diabetes and metabolic syndrome (Yaribeygi et al., 2019). Therefore, the regulation of mitochondrial ROS is of key relevance for cellular homeostasis (Kirtonia et al., 2020).

## Mitochondrion: Structure and Function

Mitochondrial dysfunction is responsible for various metabolic diseases. Therefore, targeting mitochondrial dysfunction will be a promising therapeutic strategy for metabolic disorders. Nevertheless, due to the high complexity of mitochondrial structure and function, targeting mitochondria for the treatment of metabolic disorders will be an arduous and challenging task. Mitochondrion is a double-membrane organelle with its own unique genome, containing 800 to 1000 copies of mitochondrial DNA (mtDNA) (Chen and Butow, 2005). The mitochondrion is constituted by the outer mitochondrial membrane (OMM), the intermembrane space (IMS), inner mitochondrial membrane (IMM) and mitochondrial matrix (Iacobazzi et al., 2017). Although the outer mitochondrial membrane is more permeable than the inner mitochondrial membrane, only molecules with a molecular weight of 5 kDa or less can cross the outer mitochondrial membrane due to the existence of the voltage-dependent anion channel (VDAC). The IMM and mitochondrial matrix contain various enzymes responsible for electron transport chain (ETC) and ATP generation. The tricarboxylic acid (TCA) cycle, one of the hallmark pathways in metabolism, plays an important role in the oxidation of respiratory substrates for ATP synthesis in the mitochondrial matrix (Sweetlove et al., 2010). During this process, electrons will be released and are absorbed by the ETC to produce ATP, which is the major source of cellular energy (Bhatti et al., 2017).

In IMM, complexes I (NADH ubiquinone reductase), II (Succinate dehydrogenase), III (Ubiquinol-cytochrome c reductase), and IV (Cytochrome c oxidase) constitute the ETC, and complex V is an ATP synthase (Shamsi et al., 2008). The hydrogen atoms released during the TCA cycle and fatty acid β-oxidation processes will be transferred to NAD + or FAD + to form NADH or FADH_2_ (St John et al., 2005). Then the electrons provided by NADH and FADH2 will be transferred to complex I and complex II, respectively, and then to complex III and complex IV. Meanwhile, ETC can generate electrochemical gradient by transporting protons into the intermembrane space. The electrochemical gradient serves as a source of potential energy for generating ATP in the complex V (Shamsi et al., 2008). Under physiological conditions, 0.4 to 4% of oxygen consumed by mitochondria is incompletely reduced, which will lead to the generation of ROS, and other reactive species such as nitric oxide (NO) and RNS (Bhatti et al., 2017; Marí and Colell, 2021). Despite there being multiple enzymatic and non-enzymatic antioxidant defense mechanisms in eukaryotic cells, excessive reactive species will inevitably damage mitochondrial proteins/enzymes, mitochondrial membranes and mtDNA, thereby impairing ATP generation (Bhatti et al., 2017).

## Mitochondrial Quality Control

### Mitophagy-Mediated Mitochondrial Quality Control

Considering the crucial role of mitochondria in cellular homeostasis, monitoring the quality of mitochondrial is important to avoid adverse effects (Ni et al., 2015; Pickles et al., 2018; Chan, 2020). Mitochondrial quality control can be acted in many forms including molecular, organellar and cellular levels. It’s worth noting that CAT, SOD and GSH-PX, as molecular-level oxidative stress defense mechanisms, can effectively decelerate the pace of oxidative damage to mitochondria (Naraki et al., 2021). However, the ROS-scavenging system cannot completely prevent excessive ROS-mediated damage to mitochondria. Accordingly, inhibiting the excessive production of ROS is gradually considered as a more effective way to prevent oxidative damage (Simona et al., 2019).

**FIGURE 1:**
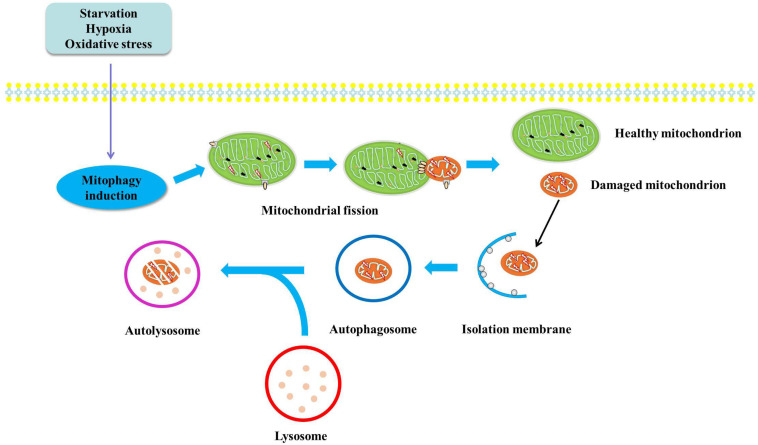
Diagram illustrates the mechanisms of mitophagy. Damaged and dysfunctional mitochondria will be segregated by autophagic membranes. And the autophagosome fuses with lysosome to form autophagolysosome, in which mitochondria will be degraded by lysosomal enzyme and the degradation products will be used as substrates for energy metabolism.

It is known that the clearance mechanism of damaged mitochondria, an important source of mitochondrial ROS, will be the potent therapeutic strategy for oxidative stress (Angajala et al., 2018; Wang R. et al., 2019). It is now generally accepted that damaged and dysfunctional mitochondria will be removed through mitophagy. In this process, damaged and dysfunctional mitochondria will be captured by autophagic membranes and further delivered to lysosomes, in which mitochondria will be degraded and the degradation products will be used as energy source for metabolism (Figure 1). With the deepening of the understanding of the physiological and pathological role of mitophagy, impaired mitophagy has been proved to be related to the development of various diseases including insulin resistance, type 2 diabetes, metabolic syndrome and non-alcoholic fatty liver disease (Su et al., 2019).

### Other Mitochondrial Quality Control Mechanisms

In addition to mitophagy-dependent mitochondrial quality control, mitochondrion also has its own regulatory mechanisms for mitochondrial quality control, such as the mitochondrial unfolded protein response (UPR^mt^) and mitochondrial fusion and fission (Roque et al., 2020).

The mitochondrial unfolded protein response (UPR^mt^) is a stress response pathway that can be activated by multiple stress conditions including mitochondrial DNA defects, decreased mitochondrial membrane potential, accumulated unfolded mitochondrial proteins and ROS detoxification (Hernando-Rodríguez and Artal-Sanz, 2018; Roque et al., 2020). At present, the molecular mechanism of UPR^mt^ in *Caenorhabditis elegans (C. elegans)* is better understood than in mammals. Activating transcription factor associated with stress (ATFS-1), a key regulatory factor for UPR^mt^ in *Caenorhabditis elegans*, has an N-terminal mitochondrial targeting sequence. Normally, ATFS-1 is imported into mitochondria and is degraded by the matrix-localized protease LON. However, in the case of mitochondrial stress such as respiratory chain dysfunction and ROS, a portion of ATFS-1 will be transferred to the nucleus to activate UPR^mt^ to regulate the abundance of mitochondrial chaperones and proteases to preserve mitochondrial homeostasis (Wrobel et al., 2015; Melber and Haynes, 2018). It’s worth noting that if mitochondrial dysfunction continues to worsen, other mitochondrial quality control mechanisms, such as mitophagy, will be activated to clear away those dysfunctional mitochondria (Ma X. et al., 2020).

Mitochondria are dynamic organelles that continuously undergo fission and fusion to maintain the balance of small fragmented mitochondria and long interconnected mitochondrial network, which is essential for cell growth, division, and distribution of mitochondria during differentiation (van der Bliek et al., 2013; Roque et al., 2020). Mitochondrial fission will split a mitochondrion into a healthy mitochondrion with increased mitochondrial membrane potential and a dysfunctional one containing diminished mitochondrial membrane potential, damaged proteins and damaged mtDNA. And then the dysfunctional mitochondrion will be targeted and degraded by mitochondrial quality control mechanisms such as mitophagy (Tahrir et al., 2019). Various regulatory factors involved in mitochondrial fission include dynamin-related protein 1 (DRP1), fission 1 (Fis1), mitochondria fission factor (Mff), mitochondrial dynamics protein of 49 kDa (MID49) and MID51 (Ni et al., 2015). When mitochondrial fission occurs, DRP1 will translocate to the outer mitochondrial membrane from cytoplasm via actin and microtubule mechanisms, and then interacts with Fis1, MFF, MID49 and MID51 to cleave mitochondrion (Anzell et al., 2018). On the other hand, mitochondrial fusion allows damaged mitochondria to fuse with healthy mitochondria to facilitate the equilibration of mitochondrial components, such as mtDNA, proteins and metabolites, thereby enhancing respiratory chain activity and maintaining mitochondrial homeostasis (Baker et al., 2011). Mitochondrial fusion is regulated by dynamin-related GTPase proteins mitofusin 1 (MFN1), mitofusin 2 (MFN2) and optic atrophy 1 (OPA1). MFN1 and MFN2 are responsible for the fusion of the outer mitochondrial membranes, and OPA1 is responsible for the fusion of the inner mitochondrial membranes (Ni et al., 2015). Therefore, outer mitochondrial membrane fusion is carried out in an MFN1/MFN2-dependent manner and inner mitochondrial membrane fusion is performed in an OPA1-dependent manner, and GTP hydrolysis provides energy for the process (Roque et al., 2020). However, when the stimulation severely interferes with mitochondria and causes the mitochondrial membrane potential to dissipate, mitochondrial fusion will be halted to prevent those mitochondria from fusing with the healthy network, thereby limiting the damage caused by dysfunctional mitochondria (Baker et al., 2011).

## Mitochondrial Dysfunction and Insulin Resistance

Insulin resistance is a pathological state in which target cells including hepatic cells, adipose cells and skeletal muscle cells are insensitive to the physiological level of insulin (Vazirani et al., 2016; Archer et al., 2018; Nishida et al., 2021). Mounting evidence reveals that insulin resistance is a common risk factor for various metabolic diseases such as metabolic syndrome, obesity, type 2 diabetes, non-alcoholic fatty liver disease and cardiovascular diseases (Patel et al., 2016; Gluvic et al., 2017; Yazıcı and Sezer, 2017; Czech, 2020; Fujii et al., 2020). Of note, a growing number of studies have established the causal relationship between mitochondrial dysfunction and insulin resistance (Ba Razzoni et al., 2012; Yazıcı and Sezer, 2017; Yaribeygi et al., 2019). Now, the complexity of mitochondrial function and the complicated relationship between mitochondrial dysfunction and the pathogenesis of insulin resistance have led to the development of many theories describing the mechanism connecting mitochondrial dysfunction and insulin resistance.

### Ectopic Lipid Accumulation and Insulin Resistance

When the causal relationship between mitochondrial dysfunction and insulin resistance was first confirmed, more and more theories describing the relationship between mitochondrial dysfunction and insulin resistance were proposed. Mitochondria are the main sites of fatty acid β-oxidation, which is the main degradation mechanism of fatty acids in cells (Su et al., 2019). Therefore, mitochondrial dysfunction will cause inefficient fatty acid oxidation, which will lead to ectopic lipid accumulation in non-adipose tissues including liver, muscle and pancreas (Martin and McGee, 2014). Ectopic lipid accumulation will result in the remarkable increase in lipid metabolites such as ceramide and diacylglycerol, which are verified to impair insulin signaling pathway and cause insulin resistance (Montgomery et al., 2019). Now, many theories describing the potential mechanism of ceramide-induced insulin resistance have been put forward (Petersen and Shulman, 2017). First, evidence shows that ceramide induces insulin resistance via suppressing ATK activation through two mechanisms: increases proteinphosphatase-2A (PP2A) and PKCζ activities, thereby disturbing AKT translocation (Petersen and Shulman, 2017). Moreover, the connection among ceramide, adipose inflammation and NLRP3 inflammasome also provides another evidence for ceramide-induced insulin resistance (Turpin et al., 2014; Xia et al., 2015; Petersen and Shulman, 2017). One putative mechanism is that diacylglycerol promotes the membrane translocation of PKCε, which in turn phosphorylates insulin receptor Thr1160 to impair insulin receptor kinase (IRK) activity, thereby inducing insulin resistance (Petersen et al., 2016). In conclusion, mitochondrial dysfunction will cause ectopic lipid accumulation, thereby resulting in the significant increase of ceramide and diacylglycerol, which will directly or indirectly suppress insulin signaling pathway to induce insulin resistance.

### Mitochondrial ROS and Insulin Resistance

Mitochondrial ROS has been recognized as the leading cause of insulin resistance. Bloch-Damti and Bashan (2005) brought forward a viewpoint that mitochondrial ROS should be accepted as a possible cause of insulin resistance in animal models of diabetes. However, these initial evidence don’t specify whether ROS that induces insulin resistance originates from mitochondria. Since mitochondria are the major sources of cellular ROS, it is necessary to explore the role of mitochondrial ROS in insulin resistance. Indeed, subsequent studies verified the causal relationship of mitochondrial ROS and insulin resistance (Anderson et al., 2009). Anderson et al. believe that mitochondrial ROS is not only an indicator of energy balance, but also a regulator of cellular redox environment, linking cellular metabolic balance with the control of insulin sensitivity (Anderson et al., 2009). There are several potential mechanisms linking mitochondrial ROS and insulin resistance. First, mitochondrial ROS activates various serine kinases that phosphorylate IRS protein and suppresses serine/threonine phosphatase activity to inhibit insulin signaling pathway (Kim et al., 2008; Fisher-Wellman and Neufer, 2012). Furthermore, mitochondrial ROS activates apoptosis signal-regulating kinase 1 (ASK1) and c-jun NH2-terminal kinases (JNK), increases serine phosphorylation of IRS-1, and decreases insulin-stimulated tyrosine phosphorylation of IRS-1, resulting in insulin resistance (Nishikawa and Araki, 2007). However, the only available experimental data has just begun to support those mechanisms, and the detailed mechanism of mitochondrial ROS-induced insulin resistance still needs further exploration.

## Mitophagy Signaling Pathways

Mitophagy, a mitochondrial quality control mechanism, selectively removes dysfunctional mitochondria to preserve mitochondrial function and maintain cellular energy homeostasis. In mammalian cells, there are three main signaling pathways that regulate mitophagy: PINK1/Parkin-dependent mitophagy, BNIP3/NIX- dependent mitophagy and FUNDC1- dependent mitophagy.

### PINK1/Parkin-Dependent Mitophagy

Lemasters et al. (1998) discovered that dysfunctional mitochondria were engulfed into autophagosome and then fused with lysosome, in which the dysfunctional mitochondria were degraded. Then Lemasters (2005) further proposed the concept “mitophagy” for the first time to describe the process of eliminating damaged mitochondria. Narendra et al. (2009) confirmed that PTEN-induced putative kinase 1 (PINK1) and the E3 ubiquitin ligase Parkin are two crucial mediators regulating mitophagy in mammalian cells. In general, PINK1 is located in the outer mitochondrial membrane, but PINK1 cannot be detected in healthy mitochondria. Because, after being located into the mitochondrial matrix, PINK1 is cleaved by intramembrane-cleaving protease PARL and then the truncated form of PINK1 is released into the cytoplasm, in which PINK1 is further degraded by the ubiquitin proteasome system to remain at a low basal level (Wu et al., 2015; Wang H. et al., 2019). However, in dysfunctional mitochondria, PINK1 cannot be transported into the inner mitochondrial membrane, thereby avoiding cleavage by intramembrane-cleaving protease PARL. Subsequently, PINK1 located in the outer mitochondrial membrane will recruit autophagy receptors including SQSTM1/p62, nuclear dot protein 52 (NDP52) and optineurin (OPTN), which can bind to LC3 to combine the dysfunctional mitochondria and autophagosomes, eventually dysfunctional mitochondria will be degraded in autolysosome (Moreira et al., 2017). To further activate mitophagy, PINK1 also phosphorylates Ser65 in the ubiquitin and ubiquitin-like domain of Parkin, and further facilitates Parkin localization from the cytosol to the outer mitochondrial membrane of dysfunctional mitochondria (Wang H. et al., 2019; Ma X. et al., 2020).

**FIGURE 2:**
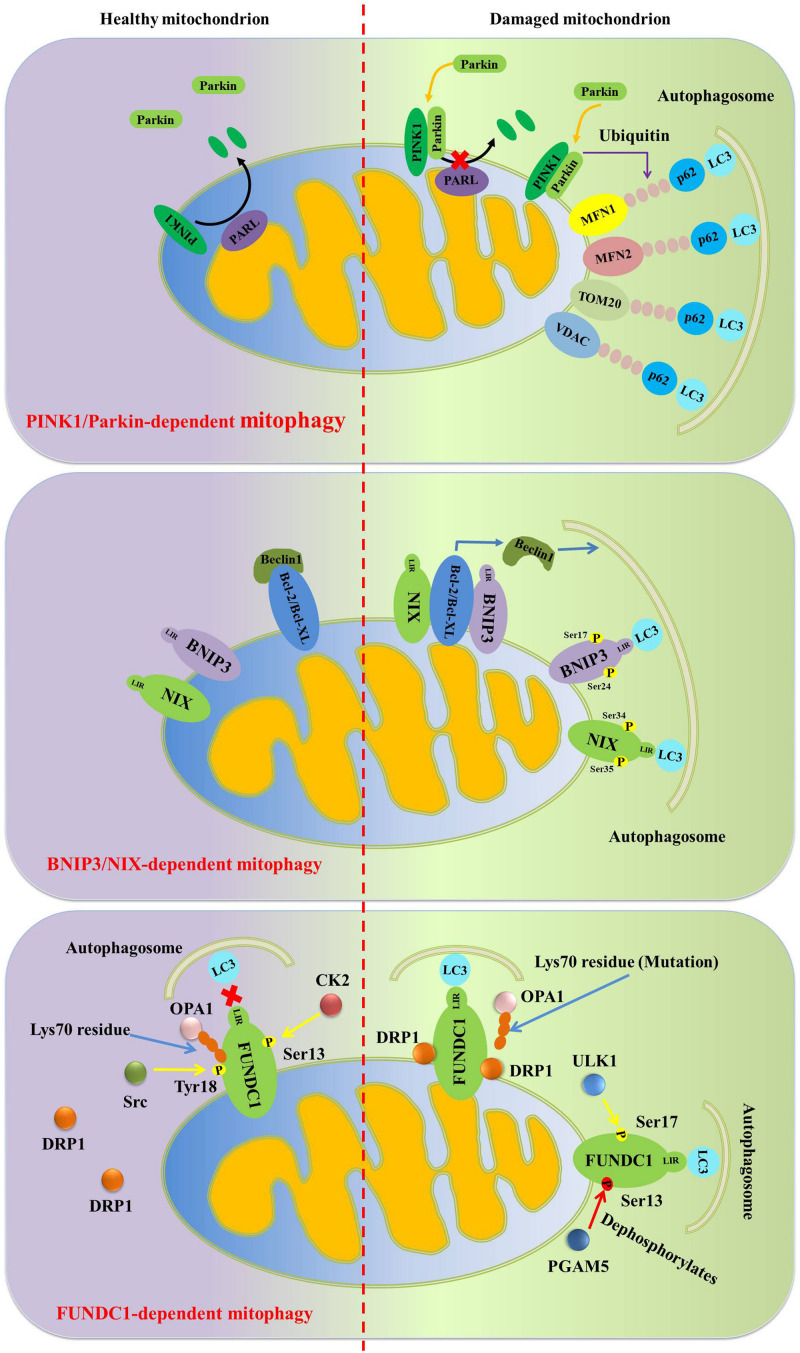
Diagram illustrates the signaling pathway regulating mitophagy. In damaged mitochondria, PINK1 located in the outer mitochondrial membrane, can phosphorylate Ser65 in the ubiquitin and ubiquitin-like domain of Parkin and further facilitates its localization from the cytosol to the outer mitochondrial membrane. Moreover, Parkin can also promote the ubiquitination of MFN1, MFN2, TOM20 and VDAC to further induce mitophagy. In response to hypoxia and nutritional deprivation, BNIP3 and NIX-mediated mitophagy is induced. In this process, BNIP3 and NIX directly interact with LC3 to enhance mitophagy. And BNIP3 and NIX also bind to the BH3 domain of Beclin1 to activate mitophagy. What’s more, similar to BNIP3/NIX, FUNDC1 interacts with LC3 through its LIR to activate mitophagy under hypoxic conditions.

Additionally, Parkin can also promote the ubiquitination of the mitochondrial fusion proteins mitofusin 1 (MFN1) and mitofusin 2 (MFN2), the mitochondrial adapter protein Miro1, translocase of outer mitochondrial membrane 20 (TOM20), and voltage-dependent anion channel (VDAC) to induce mitophagy (Nardin et al., 2016; Bradshaw et al., 2020). Normally, there are two major mechanisms involved in Parkin-dependent mitophagy: the first mechanism is that Parkin ubiquitinates MFN1 and MFN2, and further degraded by the proteasome, resulting in mitochondrial fission. Mitochondrial fission contributes to the separation of dysfunctional mitochondria from healthy network, and then dysfunctional mitochondria will be engulfed by autophagosomes and further degraded in autolysosomes (Eid et al., 2016; Ma X. et al., 2020). Additionally, Parkin-mediated the ubiquitination of mitochondrial outer membrane proteins VDAC will promote the recognition of VDAC by autophagy receptors such as histone deacetylase 6 (HDAC6) and p62. Subsequently, p62 will bind to LC3 positive autophagosomes to promote dysfunctional mitochondria to be captured by autophagosomes and then be degraded in autolysosomes (Figure 2; Moreira et al., 2017).

### BNIP3/NIX-Dependent Mitophagy

In order to cope with hostile environments such as hypoxia and nutritional deficiencies, mitochondrial fission will enhance and mitophagy activation will also increase to degrade excessive mitochondria, and finally adaptively reducing mitochondrial quantity and maintaining cellular energy homeostasis. Generally speaking, this kind of stress-induced mitophagy is mediated by BCL-2/adenovirus E1B interacting protein 3 (BNIP3) and Nip-like protein X (NIX) (Lampert et al., 2019; Xu et al., 2020). Of note, contrary to PINK1/Parkin-mediated indirect connection between damaged mitochondria and autophagosomes, BNIP3 and NIX induce mitophagy by direct connecting damaged mitochondria to autophagosomes (Moreira et al., 2017). The phosphorylation of Ser17 and Ser24 on BNIP3 can promote the affinity of BNIP3 and LC3, thereby activating mitophagy (Zhu et al., 2013). Furthermore, Beclin1 induces mitophagy in the form of Beclin1-Vps34 -Vps15 complexes (Ma et al., 2014; Wang et al., 2017). However, Bcl-2 and Bcl-XL can bind to the BH3 domain of Beclin1 to form Beclin1-Bcl-2-Bcl-XL complexes to inhibit mitophagy. It is interesting to note that BNIP3 and NIX are easier to bind to Bcl-2 and Bcl-XL than Beclin1, which release Beclin1 from Beclin1-Bcl-2-Bcl-XL complexes and subsequently induces mitophagy (M Chiara et al., 2014; Chiang et al., 2018). What’s more, rashomolog enriched in brain (Rheb) can activate the mammalian target of rapamycin (mTOR) to inhibit mitophagy, but BNIP3 can suppress Rheb/mTOR activation (Gong et al., 2018). Similar to BNIP3, NIX also can directly interact with LC3, and the phosphorylation of Ser34 and Ser35 on NIX further promotes this interaction. Moreover, Parkin-mediated NIX ubiquitination will recruit NBR1, an autophagy cargo receptor, to enhance mitophagy-mediated degradation of dysfunctional mitochondria.

Glick et al. (2012) have confirmed that elevated lipid synthesis, reduced fatty acids β-oxidation, impaired glucose tolerance, and elevated ROS levels, inflammation response and steatohepatitis are found in liver-specific BNIP3 gene knockout mice. Further research finds that elevated numbers of mitochondria but impaired mitochondrial function are also found in liver-specific BNIP3 gene knockout mice, which is characterized by loss of mitochondrial membrane potential, dysfunctional oxidative phosphorylation and reduced fatty acids β-oxidation (Glick et al., 2012; Boland et al., 2014). These results confirm the critical role of BNIP3 in maintaining mitochondrial integrity, which contributes to the prevention and treatment of metabolic diseases (Figure 2).

### FUNDC1-Dependent Mitophagy

FUN14 domain-containing 1 (FUNDC1) contains a conserved LC3-interacting region (LIR). Accumulating study confirms that FUNDC1 is a crucial hypoxia-mediated mitophagy regulator (Chen et al., 2017; Li et al., 2018; Zhang, 2020). Similar to BNIP3/NIX, FUNDC1 directly interacts with LC3 through its LIR under hypoxic conditions (Kuang et al., 2016). However, under normal conditions, protein kinase Src and CK2 will phosphorylate Tyr18 and Ser13 of FUNDC1 to disturb its interaction with LC3 (Kuang et al., 2016). Nevertheless, the serine/threonine kinase UNC-51 like kinase 1 (ULK1) can phosphorylate FUNDC1 at Ser17 to activate mitophagy under hypoxic conditions. Moreover, mitochondrial fission and fusion mediators including PGAM5, OPA1 and DRP1 can interact with FUNDC1 to regulate mitophagy. Under hypoxic conditions, the mitochondrial phosphatase PGAM5 dephosphorylates the Ser13 of FUNDC1 to promote its interaction with LC3 (Ma K. et al., 2020). PGAM5-mediated dephosphorylation of FUNDC1 also disrupts its association with mitochondrial fusion protein OPA1, thereby inhibiting mitochondrial fusion (Ma K. et al., 2020).

Under normoxic conditions, FUNDC1 is located in the endoplasmic reticulum-mitochondria contact sites. In order to cope with hypoxic stress, FUNDC1 associates with the endoplasmic reticulum (ER) protein calnexin (CANX) in mitochondria-associated ER membranes and then recruits DRP1 to promote mitochondrial fission and further activates mitophagy (Palikaras et al., 2018). Although FUNDC1, BNIP3 and NIX can directly interact with LC3 to activate mitophagy, the interaction among FUNDC1, BNIP3 and NIX is still not well explained, and their synergy is very important for mitophagy-mediated mitochondrial quality control (Figure 2).

## Phytochemicals

Phytochemicals derived from natural plants are often used to prevent and/or treat metabolic disorders due to their unique therapeutic properties and safety (Bacanli et al., 2019). Plentiful medicinal and non-medicinal natural plants have been used to treat diseases from time immemorial in the world on account of the accessibility and low cost. Studies have shown that phytochemicals such as akebia saponin D, quercetin, cyanidin-3-O-glucoside, corilagin, notoginsenoside R1, scutellarin, salvianolic acid B, resveratrol and curcumin show protective effects against metabolic diseases, and their plant origins, effects and molecular mechanisms on metabolic diseases are provided in Table 1.

**TABLE 1:** The plant origins, protective effects and mechanisms of phytochemicals on metabolic disorders.

Phytochemical	Plant origin	Health effect	Molecular mechanism	References
Akebia saponin D	*Dipsacus asper* Wall. ex Henry; *Lonicera rupicola* Hook. f. et Thoms. var. *syringantha* (Maxim.) Zabel; *Dioscorea futschauensis* Uline ex R.Kunth; *Anemone rupestris* Hook. f. et Thoms. *subsp. gelida* (Maxim.) Lauener.	• Atherosclerosis; • Hyperlipidemia; • Hepatic steatosis; • Metabolic syndrome; • Acute myocardial infarction.	• Suppressing oxidative stress; • Enhancing autophagy; • Regulating intestinal microbiota; • Enhancing mitophagy; • Suppressing intestinal barrier injury; • Scavenging lipid peroxidation and preventing mitochondrial damage.	Li et al., 2010; Gong et al., 2018; Yang S. et al., 2019; Zhou et al., 2019b; Yang et al., 2021
Quercetin	*Astragalus membranaceus(Fisch.)*Bge.; *Sophora japonica* L.; *Platycladus orientalis* (L.) Franco; *Panax notoginseng* (Burk.) F. H. Chen; *Solanum tuberosum* L.; *Hippophae rhamnoides* L.	• Type 2 diabetes mellitus; • Cardiovascular; • Non-alcoholic fatty liver disease; • Obesity; • Alcohol-induced liver injury; • Ethanol-induced liver steatosis.	• Suppressing insulin resistance; • Suppressing inflammation response and oxidative stress; • Enhancing lipid metabolism; • Enhancing hepatic VLDL assembly and lipophagy; • Suppressing chronic inflammation.	Eid and Haddad, 2017; Patel et al., 2018; Zhu et al., 2018; Yang H. et al., 2019
Cyanidin-3-O-glucoside	Black rice; Black soya bean; *Ipomoea batatas (L.)* Lam; *Lagerstroemia indica* L.; *Begonia fimbristipula* Hance.	• Diabetic nephropathy; • Non-alcoholic fatty liver disease; • Hypercholesterolemia; • Obesity; • Metabolic syndrome.	• Enhancing glutathione pool; • Regulating the secretion of adipokines from brown adipose tissue; • Enhancing LXRα-CYP7A1-bile acid excretion pathway; • Suppressing the expression of lipoprotein lipase; • Suppressing inflammation response.	Wang et al., 2012; Bhaswant et al., 2015; Pei et al., 2018; Qin et al., 2018; Li X. et al., 2020
Corilagin	*Phyllanthus urinaria* L.; *Phyllanthus emblica* L.; *Phyllanthus ussuriensis* Rupr. et Maxim.; *Phyllanthus niruri* Linn.; *Geranium wilfordii* Maxim.	• Acetaminophen-induced hepatotoxicity; • Hepatic fibrosis; • Non-alcoholic fatty liver disease; • Atherosclerosis; • Type 2 diabetes mellitus.	• Enhancing AMPK/GSK3β-Nrf2 signaling pathway; • Suppressing miR-21-regulated TGF-β1/Smad signaling pathway; • Suppressing oxidative stress and restoring autophagic flux; • Suppressing toll-like receptor-4 signaling pathway.	Lv et al., 2019; Zhang R. et al., 2019; Zhou et al., 2019c; Li Y. et al., 2020
Notoginsenoside R1	*Panax notoginseng* (Burk.) F. H. Chen.	• Diabetic retinopathy; • Chronic atrophic gastritis; • Type 2 diabetes mellitus; • Cardiac hypertrophy; • Atherosclerosis.	• Enhancing PINK1-dependent mitophagy; • Suppressing oxidative stress; • Suppressing inflammation response; • Suppressing proinflammatory monocytes; • Suppressing TLR4/NF-κB pathway; • Enhancing Nrf2-mediated HO-1 expression.	Chen et al., 2019; Luo et al., 2019; Zhang B. et al., 2019; Liu H. et al., 2020
Scutellarin	*Erigeron breviscapus* (Vant.) Hand. - Mazz.; *Scutellaria altissima* L.; *Scutellaria barbata* D. Don.	• Atherosclerosis; • Diabetic retinopathy; • Non-alcoholic fatty liver disease; • Type 2 diabetes mellitus.	• Suppressing oxidative stress-induced vascular endothelial dysfunction and endothelial cell damage; • Suppressing VEGF/ERK/FAK/Src pathway Signaling; • Suppressing oxidative stress.	Long et al., 2015; Mo et al., 2018; Zhang X. et al., 2018; Long et al., 2019
Salvianolic acid B	*Salvia miltiorrhiza* Bge.	• Diabetic cardiomyopathy; • Myocardial ischemic injury; • Endothelial dysfunction; • Obesity; • Atherosclerosis; • Non-alcoholic fatty liver disease; • Type 2 diabetes mellitus.	• Suppressing insulin-like growth factor-binding protein 3 expression; • Suppressing NLRP3 inflammasome; • Suppressing apoptosis; • Regulating gut microbiota abundances and LPS/TLR4 signaling pathway; • Suppressing YAP/TAZ/JNK signaling pathway; • Enhancing SIRT1-mediated inhibition of HMGB1; • Enhancing insulin sensitivity.	Huang et al., 2015; Zeng et al., 2015; Hu et al., 2020; Ko et al., 2020; Li C.-L. et al., 2020; Li L. et al., 2020; Yang et al., 2020
Resveratrol	*Polygonum cuspidatum* Sieb.et Zucc.; *Belamcanda chinensis*(L.)Redouté; *Smilax davidiana* A. DC.; *Reynoutria ciliinerve* C. F. Fang transl. nov.; *Ampelopsis japonica* (Thunb.) Makino; *Scirpus yagara* Ohwi.	• Metabolic syndrome; • Type 2 diabetes mellitus; • Atherosclerosis; • Non-alcoholic fatty liver disease; • Obesity; • Diabetic cardiomyopathy; • Myocardial ischemia.	• Suppressing inflammation response and oxidative stress; • Enhancing insulin sensitivity; • Enhancing mitochondrial function; • Remodeling gut microbiota; • Suppressing STIM1-mediated intracellular calcium accumulation.	Chen et al., 2016; Ma et al., 2017; de Ligt et al., 2018; Seyyedebrahimi et al., 2018; Tabrizi et al., 2018; Huang et al., 2019; Szkudelska et al., 2019; Xu et al., 2019; Huang et al., 2020
Curcumin	*Curcuma phaeocaulis* Valeton; *Curcuma longa* L.; *Radix Curcumae*.	• Metabolic syndrome; • Type 2 diabetes mellitus; • Atherosclerosis; • Non-alcoholic fatty liver disease; • Obesity; • Diabetic cardiomyopathy; • Myocardial ischemia.	• Suppressing hyperlipidemia; • Suppressing oxidative stress; • Enhancing insulin sensitivity; • Regulating intestinal barrier function; • Suppressing TLR4-related inflammation response; • Enhancing autophagy; • Suppressing cell apoptosis.	Bradford, 2013; Ghosh et al., 2018; Mokhtari-Zaer et al., 2018; Yao et al., 2018; Zhang S. et al., 2018; Ahmed et al., 2019; Azhdari et al., 2019; Pivari et al., 2019; Ren et al., 2020; Wu et al., 2020

## Phytochemical-Mediated Mitophagy in Metabolic Disorders

### Non-alcoholic Fatty Liver Disease

**FIGURE 3:**
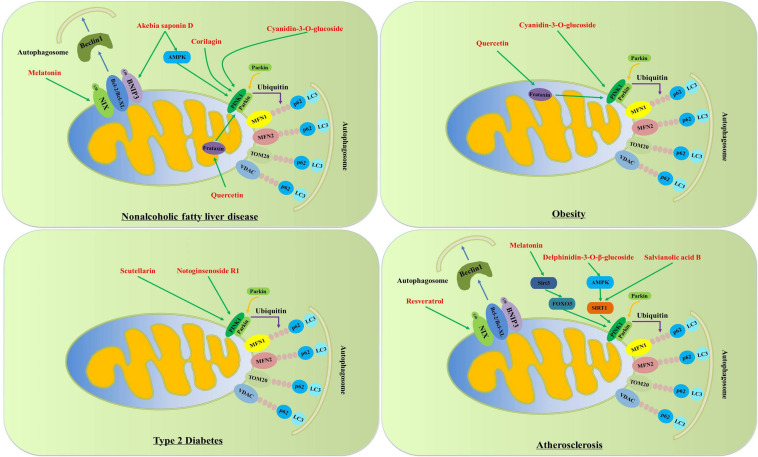
Phytochemical activates mitophagy to treat metabolic disorders. Phytochemical activates PINK1-Parkin-dependent mitophagy and BNIP3/NIX-dependent mitophagy to treat metabolic disorders. In PINK1-Parkin-dependent mitophagy, PINK1 phosphorylates Ser65 in the ubiquitin and ubiquitin-like domain of Parkin and further facilitates its localization from the cytosol to the outer mitochondrial membrane of dysfunctional mitochondria. Moreover, Parkin can further promote the ubiquitination of MFN1, MFN2, TOM20 and VDAC, which can be identified by autophagy receptors p62 and then bind to LC3 positive autophagosomes to promote dysfunctional mitochondria to be captured by autophagosomes. Additionally, BNIP3 and NIX are easier to bind to Bcl-2 and Bcl-XL than Beclin1, which causes the release of Beclin-1 from Beclin1-Bcl-2-Bcl-XL complexes and subsequently induces mitophagy.

Non-alcoholic fatty liver disease (NAFLD) refers to the accumulation of fat in the liver of an individual, which is not caused by excessive alcohol consumption (Younossi et al., 2016; Geng et al., 2021). There is a consensus that NAFLD is the risk factor of non-alcoholic steatohepatitis, cirrhosis and hepatocellular carcinoma. Currently, more and more phytochemicals have been found to target mitophagy to prevent/treat metabolic diseases. For example, akebia saponin D, the major active component of *Dipsacus asper* Wall. ex Henry, activates BNIP3-mediated mitophagy to alleviate hepatic steatosis in oleic acid-induced buffalo rat liver cells. Liu et al. have observed that quercetin, the most common flavonoid from *Astragalus membranaceus (Fisch.)* Bge., alleviates non-alcoholic fatty liver disease by enhancing PINK1/Parkin-dependent mitophagy in oleate/palmitate-induced HepG2 cells and high-fat diet-fed mice (Liu et al., 2018). Consistent with the above results, Li et al. also observe that cyanidin-3-O-glucoside inhibits hepatic oxidative stress, NLRP3 inflammasomes, hepatic lipid accumulation and improves insulin sensitivity in mice with NAFLD. In palmitic acid-induced alpha mouse liver 12 (AML-12) cells, cyanidin-3-O-glucoside also suppresses lipid accumulation, IL-1β and IL-18 levels and ROS content, and up-regulates the expressions of autophagosome formation genes including phosphatidylinositol 3-kinase catalytic subunit type 3 (PIK3C3), Beclin1, ATG5, ATG12, ATG7, transcription factor EB (TFEB) and LC3-II. Moreover, in palmitic acid-induced AML-12 cells and HepG2 cells, cyanidin-3-O-glucoside also increases the expressions of PINK1, Parkin and TOM20. In hepatocytes from NAFLD patients, cyanidin-3-O-glucoside significantly decreases triglyceride, NLRP3, Caspase-1, IL-1β, IL-18 and ROS levels, and increases the expressions of PINK1, Parkin and TOM20 proteins. Li et al. speculated that cyanidin-3-O-glucoside activated PINK1-mediated mitophagy to alleviate NAFLD. Corilagin, water-soluble tannin, is found in many herbs such as *Dimocarpus* longana, *Phyllanthus* urinaria and *Phyllanthus emblica* Linn. Corilagin markedly attenuates hepatic steatosis, which is manifested by decreased serum lipids, hepatic cholesterol and triglyceride contents, down-regulated expressions of fatty acid synthesis genes including ACC1 and SREBP-1c, decreased pro-inflammatory cytokine genes including TNF-a and IL-6, and up-regulated expressions of fatty acid oxidation genes including PPARα and ACOX1. Moreover, Corilagin can restore high-fat diet-mediated mitophagy blockage via activating the expressions of Parkin and LC3-II proteins. In line with these results, Corilagin can improve mitochondrial dysfunction, evidenced by reduced ROS and MDA levels, enhanced expressions of antioxidative enzymes including SOD, GSH-PX and CAT, increased mitochondrial membrane potential and reduced mitochondrial oxidative DNA damage. In conclusion, mitophagy plays an important role in the treatment of Corilagin in NAFLD (Zhang R. et al., 2019). Melatonin is a hormone found in bacteria, eukaryotic unicells, macroalgae, plants and fungi (Hardeland and Poeggeler, 2003). In high-fat diet-fed rats, melatonin administration can significantly alleviate mitochondrial dysfunction and NAFLD. In the setting of NAFLD, NR4A1/DNA-PKcs/p53 signal pathway can promote DRP1-dependent mitochondrial fission and suppress BNIP3-mediated mitophagy. However, melatonin can suppress NR4A1/DNA-PKcs/p53 signal pathway to restore mitophagy, thereby improving mitochondrial dysfunction in NAFLD, which is manifested by enhanced ATP production, restored mitochondrial membrane potential and improved mitochondrial respiratory function (Zhou et al., 2018). Choi et al. observe that fermented Korean red ginseng extract (RG) administration remarkably improves the high-fat diet-induced hepatic steatosis, liver injury and inflammation in the setting of NAFLD. And RG also inhibits lipid accumulation in the palmitate-induced primary hepatocytes. Mechanistically, RG inhibits the activation of mTORC1 to activate mitophagy and PPARα signaling pathway to improve NAFLD (Table 2 and Figure 3; Choi et al., 2019).

**TABLE 2:** Phytochemical enhances mitophagy to treat metabolic disorders.

**Phytochemical**	**Disease**	**Type of mitophagy**	**Molecular mechanism**	**References**
Akebia saponin D	Non-alcoholic fatty liver disease	BNIP3-mediated mitophagy	• Enhancing autophagy**:** p-AMPK ↑, p-mTOR ↓ and LC3-II ↑; • Enhancing mitophagy**:** BNIP3 ↑.	Gong et al., 2018
Quercetin	Non-alcoholic fatty liver disease	PINK1/Parkin-mediated mitophagy	• Suppressing hyperlipidemia**:** triglyceride↓ and cholesterol ↓; • Suppressing lipogenic gene expression**:** fatty acid synthase (FAS) ↓; • Enhancing β-oxidation enzyme**:** carnitine palmitoyltransferase I (CPT1) ↑; • Enhancing mitochondrial function**:** respiratory control ratio ↑ and mitochondrial membrane potential ↑; • Enhancing mitophagy**:** Frataxin ↑, Parkin ↑;PINK1 ↑, Beclin1 ↑, LC3-II ↑, p62 ↓, CISD1 ↓, VDAC1 ↑, TOM20 ↓ and HIF-1α↓.	Liu et al., 2018
Cyanidin-3-O-glucoside	Non-alcoholic fatty liver disease	PINK1-mediated mitophagy	• Suppressing hyperlipidemia**:** cholesterol synthesis-related genes (HMGCR) ↓, fatty acid uptake related genes (FABP1 ↓, FATB1 ↓ and CD36 ↓), fatty acid synthesis related genes (FAS ↓, ACCα↓, SREBF1 ↓ and PPAR-γ↓), cholesterol efflux-related genes (CYP7A1 ↑) and fatty acid β-oxidation-related genes (PPARA ↑, CPT1A ↑, ACOX1 ↑ and MCAD ↑); • Suppressing inflammation**:** IL-1β↓, IL-18 ↓, NLRP3 ↓, Caspase-1 ↓, Pro-Caspase-1 ↓ and IL-1β↓; • Suppressing oxidative stress: H_2_O_2_ ↓, MDA ↓, SOD ↑, CAT ↑, GSH-PX ↑ and ROS ↓; • Enhancing mitochondrial function**:** peroxisome proliferative activated receptor- γ (NR1C3) ↑, nuclear respiratory factor 1 (NRF1) ↑, nuclear factor erythroid derived 2 like 2 (NRF2) ↑ and mitochondrial transcription factor A (TFAM) ↑; • Enhancing mitophagy**:** PINK1 ↑, Parkin ↑;LC3-II ↑, p62 ↓, TOM20 ↓, PIK3C3 ↑, Beclin1 ↑, ATG5 ↑, ATG12 ↑, ATG7 ↑ and TFEB ↑.	Li X. et al., 2020
Corilagin	Non-alcoholic fatty liver disease	Parkin-mediated mitophagy	• Suppressing hyperlipidemia**:** triglyceride ↓, cholesterol ↓, low-density lipoprotein cholesterol ↓, high-density lipoprotein cholesterol ↑; fatty acid synthesis genes (FASN ↓, ACC1 ↓, and SREBP-1c ↓) and fatty acid oxidation genes (PPARA ↑, CPT1A ↑, and ACOX1 ↑); • Suppressing inflammation**:** MCP1 ↓, F4/80 ↓, TNF-α↓ and IL-6 ↓; • Enhancing mitophagy**:** LC3-II ↑, p62 ↓, Parkin ↑ and VDAC1 ↑; • Suppressing oxidative stress**:** ROS ↓, SOD ↑, GSH-PX ↑, CAT ↑, and MDA ↓; • Enhancing mitochondrial function**:** mitochondrial membrane potential ↑ and mitochondrial biogenesis related gene (NRF1 ↑, NRF2 ↑, and TFAM ↑).	Zhang R. et al., 2019
Melatonin	Non-alcoholic fatty liver disease	BNIP3-mediated mitophagy	• Suppressing hyperlipidemia**:** triglycerides ↓ and cholesterol ↓; • Suppressing inflammation**:** IL-6 ↓, TNF-α↓ and TGF-β↓; • Suppressing oxidative stress**:** ROS ↓, SOD ↑, GSH-PX ↑ and MDA ↓; • Enhancing mitochondrial function**:** ATP generation ↑ and mitochondrial respiratory function ↑; • Enhancing mitophagy**:** DRP ↓, BNIP3 ↑, LC3-II ↑, Beclin1 ↑, Atg5 ↑, DNA-PKcs ↓, p53 ↓ and NR4A1 ↑.	Zhou et al., 2018
Cyanidin-3-O-glucoside	Obesity	PINK1-mediated mitophagy	• Suppressing cholesterol synthesis-related genes (HMGCR ↓), fatty acid uptake related genes (FABP1 ↓, FATB1 ↓ and CD36 ↓) and fatty acid synthesis related genes (FAS ↓, ACCα↓, SREBF1 ↓ and PPAR-γ↓); • Enhancing cholesterol efflux-related genes (CYP7A1 ↑) and fatty acid β-oxidation-related genes (PPARA ↑, CPT1A ↑, ACOX1 ↑ and MCAD ↑); • Suppressing inflammation response**:** IL-1β↓, IL-18 ↓, NLRP3 ↓, Caspase-1 ↓, Pro-Caspase-1 ↓ and IL-1β↓; • Suppressing oxidative stress**:** H_2_O_2_ ↓, MDA ↓, SOD ↑, l-cysteine:2-oxoglutarate aminotransferase (CAT) ↑ and glutathione peroxidase (GSH-PX) ↑ and ROS ↓; • Enhancing mitochondrial function**:** peroxisome proliferative activated receptor- γ (NR1C3) ↑, nuclear respiratory factor 1 (NRF1) ↑, nuclear factor erythroid derived 2 like 2 (NRF2) ↑ and mitochondrial transcription factor A (TFAM) ↑; • Enhancing mitophagy**:** PINK1 ↑, Parkin ↑;LC3-II ↑, p62 ↓, TOM20 ↓, PIK3C3 ↑, Beclin 1 ↑, ATG5 ↑, ATG12 ↑, ATG7 ↑ and TFEB ↑.	Li X. et al., 2020
Quercetin	Obesity	PINK1/Parkin-mediated mitophagy	• Suppressing hyperlipidemia**:** triglyceride ↓ and cholesterol ↓; • Suppressing lipogenic gene expression**:** fatty acid synthase (FAS) ↓; • Enhancing β-oxidation enzyme**:** carnitine palmitoyltransferase I (CPT1) ↑; • Enhancing mitochondrial function**:** respiratory control ratio and mitochondrial membrane potential ↑; • Enhancing mitophagy**:** Frataxin ↑, Parkin ↑, PINK1 ↑, Beclin1 ↑, LC3-II ↑, p62 ↓, CISD1 ↓, VDAC1 ↑, TOM20 ↓ and HIF-1α↓.	Liu et al., 2018
Notoginsenoside R1	Diabetic retinopathy	PINK1-mediated mitophagy	• Suppressing oxidative stress**:** ROS ↓, 4-HNE ↓, protein carbonyl ↓ and 8-OHdG ↓; • Suppressing inflammation**:** MCP-1 ↓, TNF-α↓, IL-6 ↓ and ICAM-1 ↓; • Enhancing mitophagy**:** Parkin ↑, PINK1 ↑, LC3 ↑ and p62 ↓.	Zhou et al., 2019a
Scutellarin	Diabetes-related vascular disease	PINK1/Parkin-mediated mitophagy	• Suppressing oxidative stress**:** ROS ↓, SOD ↑ and SOD2 ↑; • Enhancing mitophagy**:** LC3-II ↑, p62 ↓, Beclin1 ↑, Atg5 ↑, Parkin ↑, PINK1 ↑ and MFN2 ↑; • Suppressing vascular endothelial cell apoptosis**:** Bcl-2 ↑, Bax ↓, Cytochrome C ↓ and cleaved caspase-3 ↓.	Xi et al., 2021
Delphinidin-3-O-β-glucoside	Atherosclerosis	AMPK/SIRT1-dependent mitophagy	• Enhancing mitophagy**:** SIRT1 ↑, Phospho-AMPKα↑, LC3-II ↑ and p62 ↓.	Jin et al., 2014
Salvianolic acid B	Atherosclerosis	SIRT1-mediated mitophagy	• Suppressing inflammation**:** NLRP3 ↓, IL-1β↓, apoptosis-associated speck-like protein (ASC) ↓ and caspase-1 ↓; • Enhancing mitophagy**:** SIRT1 ↑, Parkin ↑, Beclin1 ↑, PINK1 ↓, LC3-II ↑ and p62 ↓; • Enhancing mitochondrial function**:** ROS ↓ and mitochondrial membrane potential ↑.	Hu et al., 2020
Melatonin	Atherosclerosis	Sirt3/FOXO3/Parkin-mediated mitophagy	• Suppressing inflammation**:** NLRP3 ↓, caspase-1 ↓ and IL-1β↓; • Enhancing mitophagy**:** Sirt3 ↑, FOXO3a ↓, LC3-II ↑, TOM20 ↓, Parkin ↑ and Beclin1 ↑; • Enhancing mitochondrial function**:** ROS ↓ and mitochondrial membrane potential ↑.	Ma et al., 2018
Resveratrol	Atherosclerosis	BNIP3-related mitophagy	• Suppressing oxidative stress**:** SOD ↑, GSH ↑ and GSH-PX ↑; • Enhancing mitochondrial function**:** Mitochondrial respiration complex I and III ↑; • Enhancing mitophagy**:** BNIP3 ↑, Beclin1 ↑, Atg5 ↑, HIF1 ↑ and AMPK ↑.	Li C. et al., 2020
				

### Obesity

In 2020, the last report of World Health Organization (WHO) categorizes 1.9 billion adults as overweight and more than 650 million as obese worldwide. Accumulating evidence confirms that mitophagy plays an important role in the regulation of mitochondrial content and function in the setting of obesity. In Parkin^–/–^ mice, Gouspillou et al. show that Parkin ablation causes decreased mitochondrial respiration and increased ROS production in skeletal muscles (Gouspillou et al., 2018; Pileggi et al., 2021). In liver-specific Parkin knockout mice, hepatic steatosis increased by 45% compared with wild-type mice (Edmunds et al., 2020). Although there were no differences in the number of mitochondria in liver between wild-type mice and liver-specific Parkin knockout mice, mitochondrial respiratory were obviously decreased in liver-specific Parkin knockout mice. And whole-body insulin resistance and hepatic insulin resistance were observed in high-fat diet-fed liver-specific Parkin knockout mice (Edmunds et al., 2020). In high-fat diet-fed mice, cyanidin-3-O-glucoside, a kind of anthocyanins mainly found in black rice, black beans and purple potatoes, significantly reduces body weight by activating PINK1-mediated mitophagy (Li X. et al., 2020). Quercetin is proved to inhibit the body weight gain in high-fat diet-fed mice, simultaneously suppress the level of hepatic or serum cholesterol and triglyceride, and inhibit the expression of lipogenic gene fatty acid synthase (FAS) (Liu et al., 2018). Quercetin also prevents oleate/palmitate-induced lipid accumulation in HepG2 cells (Liu et al., 2018). Mechanistically, Quercetin activates PINK1/Parkin-dependent mitophagy to improve lipid metabolism in the setting of obesity. Since obesity is considered to be a concomitant symptom of various diseases such as metabolic syndrome and NAFLD, only a few studies have focused on it. Therefore, it will be very meaningful to clarify the role of mitophagy-mediated mitochondrial quality control in the prevention and treatment of obesity in future studies (Table 2 and Figure 3).

### Type 2 Diabetes

Growing evidence has proved that mitochondrial dysfunction is a risk factor of type 2 diabetes and its complications such as hyperglycemia, diabetic nephropathy and diabetic retinopathy (Rovira-Llopis et al., 2017). Therefore, targeting mitophagy is a promising pharmaceutical strategy for type 2 diabetes and its complications. The *Astragalus mongholicus* Bunge and *Panax* notoginseng (Burk.) F.H. Chen formula (APF) consists of *Astragalus mongholicus* Bunge, *Panax* notoginseng (Burkill) F.H. Chen, *Angelica sinensis* (Oliv.) Diels, *Achyranthes bidentata* Blume, and *Ecklonia kurome* Okamura. APF significantly improves the blood urea nitrogen, serum creatinine and 24-h albuminuria of mice with diabetic nephropathy, and prevents inflammatory response in high glucose-induced renal mesangial cells and the kidney tissue of mice with diabetic nephropathy. Furthermore, increased expressions of PINK1, Parkin, Beclin1 and LC3-II are recorded in renal mesangial cells and the kidney tissue of mice with diabetic nephropathy. These results remind that APF activates PINK1/Parkin-mediated mitophagy to protect the kidney from inflammatory injury in the setting of diabetes mellitus (Wen et al., 2020). Diabetic retinopathy is a serious complication of diabetes and remains the leading cause of blindness worldwide (Zhou et al., 2019a). Notoginsenoside R1, a saponin from *Panax notoginseng*, is proved to prevent oxidative stress and inflammatory response in high glucose-treated rat retinal Müller cells and the retinas of diabetic *db/db* mice. Mechanistically, Notoginsenoside R1 improves diabetic retinopathy through activating PINK1-mediated mitophagy (Zhou et al., 2019a). There is a consensus that endothelial cell injury is a critical pathophysiological basis of diabetes-related vascular disease. Scutellarin, a main composition of *Scutellaria baicalensis* Georgi, inhibits mitochondrial-mediated apoptosis to increase cell viability of high glucose-induced human umbilical vein endothelial cells (HUVECs). Moreover, scutellarin can activate PINK1/Parkin-mediated mitophagy to improve mitochondrial function characterized by reduced ROS production, enhanced mitochondrial membrane potential and increased SOD activity. Many mechanisms involved in this effect include LC3-II, Atg5, P62, PINK1, Parkin, and MFN2. However, the effect is weakened by PINK1 gene knockdown. These results remind that scutellarin suppresses vascular endothelial cell damage caused by hyperglycemia through activating PINK1/Parkin-dependent mitophagy (Table 2 and Figure 3; Xi et al., 2021).

### Cardiovascular Disease

Cardiovascular disease is the leading cause of death in the world, including cardiac failure, coronary heart disease, myocardial infarction and atherosclerosis (Mir et al., 2021). Atherosclerosis is one of the most common causes of cardiovascular disease (Poznyak et al., 2021). It is known that mitochondrial dysfunction is an important cause of inflammatory response, lipid accumulation and oxidative stress, which are the pathogenic factors of atherosclerosis (Poznyak et al., 2021). Given the critical role of mitophagy in maintaining mitochondrial function in cardiovascular disease, the regulatory mechanism of mitophagy has captured the attention of researchers around the world. Among various pathogenic factors, vascular endothelial injury is the driving force for the development of atherosclerosis, and oxidized low-density lipoprotein (ox-LDL) plays a key role in this process (Fang et al., 2014). Delphinidin-3-O-β-glucoside, a natural anthocyanin, is abundant in black soybean, bilberries and cereals (Park et al., 2019). Jin et al. have demonstrated that delphinidin-3-O-β-glucoside suppresses ox-LDL-induced cell death in human umbilical vein endothelial cells. Further studies have suggested that activated AMPK/SIRT1-dependent mitophagy is the pivotal molecular mechanism for the protective effect of delphinidin-3-O-β-glucoside on human umbilical vein endothelial cells (Jin et al., 2014). Myocardial ischemic injury, a common kind of cardiovascular disease, can cause irreversible damage to heart. It is known that NLRP3 inflammasome-mediated inflammatory response is the pivotal mechanism of the development of myocardial ischemic injury (Zhu et al., 2015; Yao et al., 2020). Salvianolic acid B, an active constituent of *Salvia miltiorrhiza* Bge, significantly prevents acute myocardial ischemic injury in rats with isoproterenol-induced acute myocardial ischemia. In lipopolysaccharide + adenosine triphosphate-administrated H9C2 cells, salvianolic acid B markedly prevents ROS production, NLRP3 inflammasome-mediated inflammatory response and cell apoptosis, and enhances mitochondrial membrane potential. Further evidence has confirmed that salvianolic acid B promotes SIRT1-mediated mitophagy to restore mitochondrial function, thereby preventing myocardial ischemic injury (Hu et al., 2020). Accumulating evidence has demonstrated that NLRP3 inflammasome-regulated inflammatory responses are responsible for the development of atherosclerosis. Recent evidence reminds that mitochondrion is a critical regulator for inflammatory responses because mitochondrial ROS is considered as a potent activator of NLRP3 inflammasome (Gurung et al., 2015; Jin et al., 2017). It is known that mitophagy plays a pivotal role in maintaining mitochondrial function though selectively eliminating dysfunctional mitochondria. Therefore, mitophagy is primarily considered as a regulator for NLRP3 inflammasome-mediated inflammatory response through scavenging mitochondrial ROS (Zhou et al., 2011; Kim et al., 2016). Ma et al. verify that melatonin activates Sirt3/FOXO3/Parkin-mediated mitophagy to scavenge excessive mitochondrial ROS, thereby suppressing NLRP3 inflammasome activation and then ameliorating atherosclerosis (Ma et al., 2018). Chen et al. also confirm that melatonin protects vascular smooth muscle cells against calcification by promoting AMPK/OPA1-dependent mitophagy. In ox-LDL-administrated human umbilical vein endothelial cells, cell apoptosis, cell proliferation arrest, impaired mitochondrial respiration, excessive mitochondrial ROS and mitochondrial dysfunction are observed. However, resveratrol, a potent natural antioxidant from *Vitis vinifera* L., enhances the expressions of hypoxia-inducible factor-1 (HIF-1) and AMPK protein to activate BNIP3-related mitophagy, thereby promoting mitochondrial respiration, scavenging excessive mitochondrial ROS and favoring endothelial cell survival (Li C. et al., 2020). The increased senescence of vascular endothelial cells evoked by high glucose and palmitate will cause endothelial dysfunction, leading to diabetic cardiovascular complications. The traditional Chinese medicine Ginseng-Sanqi-Chuanxiong (GSC) is composed of *Panax ginseng* C. A. Mey., *Panax notoginseng* (Burk.) F. H. Chen, and *Ligusticum chuanxiong* Hort. at a ratio of 2:3:4. Wang et al. find that GSC extract inhibits high glucose and palmitate-induced vascular endothelial cell senescence via activating AMPK-dependent mitophagy to suppress mitochondrial ROS production (Table 2 and Figure 3; Wang et al., 2020).

At present, more and more clinical trials have confirmed the efficacies and mechanisms of phytochemicals for metabolic diseases, such as quercetin, melatonin and resveratrol (Supplementary Table 1). However, as more and more phytochemicals have been used to prevent or treat metabolic disorders, their side effects should also be taken seriously. For example, high-dose of quercetin can cause liver damage in an animal model of hyperhomocysteinemia (Meng, 2013). High concentration of resveratrol (50 μM) suppresses the cell viability of transformed macrophages and carcinoma cells. However, low concentration of resveratrol (5 μM) promotes the cell viability of these cells (Shaito et al., 2020). Moreover, low concentration of resveratrol (0.5–5μM) has no significant effect on the viability or function of rat pancreatic cells, while high concentration of resveratrol (50 μM) increases pancreatic cell apoptosis (Shaito et al., 2020). Surprisingly, resveratrol can stimulate oxidative stress to induce mitochondrial-mediated cancer cell apoptosis (Ashrafizadeh et al., 2020). In conclusion, exploring the optimal phytochemical dosage and the different molecular mechanisms shown in different disease models can maximize its health benefits without causing toxicity problems, which will be an area worthy of in-depth research.

## Summary and Future Perspectives

**FIGURE 4:**
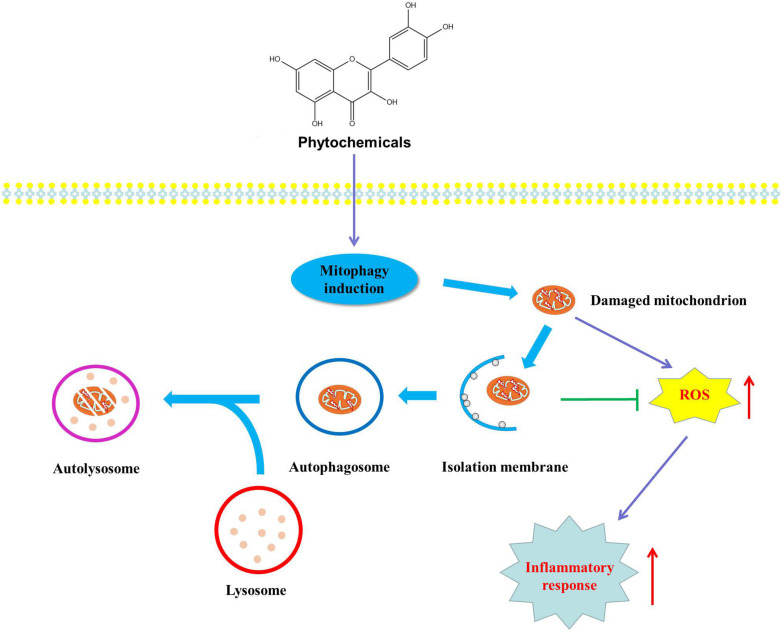
Mechanisms of phytochemical-mediated mitophagy in the treatment of metabolic disorders. Phytochemical activates mitophagy to degrade damaged mitochondria to prevent the production of mitochondrial ROS which can trigger oxidative stress and inflammatory response, and eventually preventing and treating metabolic diseases.

Mitochondrion is an important organelle responsible for various cellular processes including ATP generation, energy metabolism, ROS production and Ca^2+^ homeostasis, cell survival and death. Accumulating evidence proves that mitochondrial dysfunction is involved in various metabolic disorders such as NAFLD, obesity, type 2 diabetes and cardiovascular disease. Mitophagy, a major mechanism of mitochondrial quality control, can selectively degrade dysfunctional mitochondria to maintain mitochondrial integrity and function. Generally speaking, mitophagy selectively degrades damaged mitochondria to suppress damaged mitochondria-derived ROS which will significantly damage healthy mitochondria and ultimately resulting in mitochondrial dysfunction. Despite increasing evidence has greatly improved our understanding of the underlying mechanisms involved in the regulation of mitophagy in metabolic disorders, some adverse results are also reported. Montgomery et al. consider that mitophagy-mediated elimination of damaged mitochondria inevitably decreases mitochondrial number, leading to decreased substrate oxidation, and finally impairing mitochondrial function (Montgomery and Turner, 2015). Now the association between mitophagy and mitochondrial homeostasis is still not fully understood, in part due to various methods for detecting mitochondrial function, different cell models and disease states (Chow et al., 2010; Montgomery and Turner, 2015). Fortunately, current studies have demonstrated that activated mitophagy will prevent damaged mitochondria-derived ROS triggering oxidative stress and inflammatory response, which are the leading causes of metabolic diseases. However, the research focused on the role of phytochemical-mediated mitophagy in the prevention and treatment of metabolic diseases is limited, and the definite relationship between phytochemical-regulated mitophagy and the treatment of metabolic diseases still needs further experimental confirmation. One thing is for sure, looking for natural compounds with mitophagic activities will provide new insights into the therapeutic intervention for mitochondrial dysfunction-related metabolic diseases (Figure 4).

## Author Contributions

ZS, YG, XH, GZ, and YZ conceived of the topic for the review. All authors listed have made a substantial direct and intellectual contribution to the work, and approved it for publication.

## Conflict of Interest

The authors declare that the research was conducted in the absence of any commercial or financial relationships that could be construed as a potential conflict of interest.

## Publisher’s Note

All claims expressed in this article are solely those of the authors and do not necessarily represent those of their affiliated organizations, or those of the publisher, the editors and the reviewers. Any product that may be evaluated in this article, or claim that may be made by its manufacturer, is not guaranteed or endorsed by the publisher.
