# Supplementary material for: Phytochemicals: Targeting Mitophagy to Treat Metabolic Disorders
Source: Front Cell Dev Biol. 2021 Aug 3;9:686820. doi: 10.3389/fcell.2021.686820 (PMC8369426; doi:10.3389/fcell.2021.686820)
Supplement: Supplementary file 1 [file Table_1.docx]

**Supplementary Table 1. The clinical trials of phytochemicals**

| **Phytochemical** | **Disease** | **Dose** | **Molecular mechanism** | **Reference** |
| --- | --- | --- | --- | --- |
| Quercetin | • Beta-thalassemia major patients receiving desferrioxamine (n=84) | • 500 mg/day for 3 months | • Improving liver function, but not oxidative stress. | (Sajadi Hezaveh et al., 2019) |
|  | • Pre-hyperuricaemic males (n=22) | • 500 mg/day for 4 weeks | • Lowering plasma uric acid concentrations without affecting fasting glucose, urinary excretion of uric acid and blood pressure. | (Shi and Williamson, 2016) |
|  | • Healthy males (n=22) | • 500 mg/day for 4 weeks | • Lowering plasma uric acid concentrations. | (Shi and Williamson, 2016) |
|  | • Post-myocardial infarction patients (n=88) | • 500 mg/day for 8 weeks | • Enhancing total antioxidant capacity and improving the insecurity dimension of quality of life, but failed to show any significant effect on inflammatory factors, blood pressure and other quality of life dimensions. | (Dehghani et al., 2021) |
|  | • Men and women with prehypertension (n=19) and stage 1 hypertension (n=22) | • 730 mg/day for 28 day | • Blood pressure was not altered in prehypertensive patients, but reductions in systolic, diastolic, and mean arterial pressures in stage 1 hypertensive patients. | (Edwards et al., 2007) |
|  | • Overweight subjects with a high-cardiovascular disease risk phenotype (n=93) | • 150 mg/day for 6 weeks | • Decreasing systolic blood pressure;  • Decreasing serum HDL-cholesterol level, while total cholesterol, TAG and the LDL:HDL-cholesterol and TAG:HDL-cholesterol ratios were unaltered;  • Decreasing plasma concentrations of atherogenic oxidised LDL, but did not affect TNF-α and C-reactive protein. | (Egert et al., 2009) |
|  | • Patients with metabolic syndrome (n=93) | • 150 mg/day for 6 weeks | • Decreasing systolic blood pressure;  • Decreasing serum HDL-cholesterol and apoA1;  • Increasing the LDL:HDL-cholesterol ratio;  • Decreasing plasma oxidized LDL and TNF-α. | (Egert et al., 2010) |
|  | • Women with Type 2 Diabetes (n=72) | • 500 mg/day for 10 weeks | • Decreasing systolic blood pressure without significant changes in diastolic blood pressure;  • Decreasing high-density lipoprotein cholesterol without significant changes in total cholesterol, low-density lipoprotein cholesterol (LDL-C), triglycerides (TG) and ratio of TG/HDL-C and LDL-C/HDL-C;  • Decreasing serum TNF-α and IL-6 levels; | (Zahedi et al., 2013) |
| Melatonin | • Healthy women (n=21) | • 5 mg/day for 4 non-consecutive days. | • Impairs glucose tolerance. | (Rubio-Sastre et al., 2014) |
|  | • Healthy volunteers (n=33) and patients with metabolic syndrome (n=30) | • 5 mg/day, 2 hour before bedtime for 2 months | • Increasing antioxidative defense (increase in CAT activity, decrease in thiobarbituric acid reactive substrates level);  • Decreasing LDL-C;  • Decreasing blood pressure; | (Koziróg et al., 2011) |
|  | • Patients with nonalcoholic fatty liver disease (n=45) | • 6 mg/day, 1 hour before bedtime for 12 weeks | • Decreasing body weight, waist circumference and abdominal circumference;  • Decreasing systolic and diastolic blood pressure;  • Decreasing Serum leptin, hs-CRP, alanine aminotransferase and aspartate aminotransferase levels;  • Decreasing the grade of fatty liver. | (Bahrami et al., 2020) |
|  | • Diabetic hemodialysis patients (n=30) and placebo (n=30) | • 10 mg/day, 1 hour before bedtime for 12 weeks | • Decreasing Pittsburgh Sleep Quality Index, Beck Depression Inventory index, and Beck Anxiety Inventory index;  • Decreasing fasting plasma glucose, serum insulin levels, and homeostasis model of assessment-insulin resistance;  • Increasing the quantitative insulin sensitivity check index;  • Decreasing serum high sensitivity C-reactive protein and plasma malondialdehyde;  • Increasing plasma total antioxidant capacity and nitric oxide levels. | (Ostadmohammadi et al., 2020) |
| Resveratrol | • Overweight patients with nonalcoholic fatty liver disease were randomised 1:1 to placebo (n=14) or resveratrol (n=14). | • 1.5 g/day for 6 months | • Not difference in plasma markers of liver injury;  • Not difference in liver lipid content;  • Not difference in histological features;  • Not difference in insulin sensitivity or markers of the metabolic syndrome. | (Heebøll et al., 2016) |
|  | • Group A (n=46): stage I hypertension (systolic blood pressure, 140-159 mmHg; diastolic blood pressure, 90-99 mmHg) and Group B (n=51): stage II hypertension (systolic blood pressure, 160-179 mmHg; diastolic blood pressure, 100-109 mmHg). | Each group was divided into two subgroups: A1 and B1, patients treated with standard antihypertensive therapy (A1, 10 mg Dapril; B1, 20 mg Dapril), and A2 and B2, patients treated with antihypertensive therapy (Dapril) plus 50 mg resveratrol for 6 months. | • Decreasing blood pressure to normal levels;  • Decreasing serum glutamate pyruvate transaminase and gamma glutamyl transferase. | (Theodotou et al., 2017) |
|  | • Obese but otherwise healthy men (n=24) | • 500 mg/day for 4 weeks | • Not difference in insulin sensitivity;  • Not difference in endogenous glucose production;  • Not difference in the turnover and oxidation rates of glucose;  • Not difference in blood pressure;  • Not difference in resting energy expenditure;  • Not difference in oxidation rates of lipid;  • Not difference in ectopic or visceral fat content;  • Not difference in inflammatory and metabolic biomarkers. | (Poulsen et al., 2013) |
|  | • Patients with type 2 diabetes and albuminuria were randomly assigned to receive either resveratrol (n=30) or placebo (n=30). | • 500 mg/day for 90 days | • Decreasing mean urine albumin/creatinine ratio;  • Not difference in eGFR and serum creatinine;  • Increasing serum antioxidant enzymes;  • Decreasing urinary albumin excretion. | (Sattarinezhad et al., 2019) |
|  | • Patients with T2D randomly were assigned to resveratrol (n=24) or placebo (n=24). | • 800 mg/day for 2 months | • Decreasing plasma protein carbonyl content and PBMCs O_2_^-•^ level;  • Increasing plasma total antioxidant capacity and total thiol content;  • Increasing Nrf2 and SOD expressions;  • Decreasing weight, BMI, and blood pressure levels;  • Not difference in the metabolic and anthropometric parameters. | (Seyyedebrahimi et al., 2018) |
|  | • Obese men (n=11). | •150 mg/day for 30 days | • Decreasing sleeping and resting metabolic rate;  • Increasing AMPK, increased SIRT1 and PGC-1α protein levels, increased citrate synthase activity without change in mitochondrial content;  • Improving muscle mitochondrial respiration on a fatty acid-derived substrate;  • Increasing intramyocellular lipid levels;  • Decreasing intrahepatic lipid content, circulating glucose, triglycerides, alanine-aminotransferase, and inflammation markers;  • Decreasing systolic blood pressure and HOMA index;  • Decreasing postprandial adipose tissue lipolysis and plasma fatty acid and glycerol. | (Timmers et al., 2011) |
|  | • Post-infarction Caucasian patients (n=40) | •10 mg/day for 3 months | • Improving left ventricular diastolic function;  • Not difference in left ventricular ejection fraction;  • Improving endothelial function;  • Decreasing low-density lipoprotein (LDL) level;  • Preventing decreased red blood cell deformability and increased platelet aggregation. | (Magyar et al., 2012) |
